# Clinical teaching in applied health science: effects of a student-led journal club on aging and physical activity

**DOI:** 10.1186/s12909-023-04732-0

**Published:** 2023-10-11

**Authors:** Tim Fleiner, Rieke Trumpf, Thiemo Schnorr, Theresa Buchner, Peter Haussermann, Wiebren Zijlstra, Tobias Morat

**Affiliations:** 1 Institute of Movement and Sport Gerontology, German Sport University Cologne, Cologne, Germany; 2 Department of Geriatric Psychiatry and Psychotherapy, LVR-Hospital Cologne, Wilhelm-Griesinger Straße 23, 51109 Cologne, Germany; 3 Institute for Geriatric Research, Ulm University Medical Center, Ulm, Germany; 4 Geriatric Center, Agaplesion Bethanien Hospital Heidelberg, Heidelberg University Hospital, Heidelberg, Germany

**Keywords:** Clinicalteaching, Journalclub, Scientificcommunication, Studscicomm, Sportandmovementgerontology

## Abstract

**Background:**

This project aims to investigate the effects of a student-led journal club on students’ critical thinking and clinical application skills in the academic field of aging and physical activity.

**Methods:**

A pre-post design analysis with data collected in four successive cohorts of the program M.Sc. Sport and Movement Gerontology was conducted. Each student assigned himself/herself to a study, and then led the journal club discussion and published a summary of the journal club via graphical abstract on social media. The students rated their perceived confidence in the beginning (T0) and after the semester (T1) via questionnaire and 5-point Likert scales addressing their ability to review and summarize the evidence, to present it in a journal club and to lead the discussion.

**Results:**

41 students (32 women, M = 25 years SD 1.9 years) were included. The journal club was rated as “very good” (median 2, IQR 1). Students’ confidence on participating, leading the journal club and transferring the results into clinical practice improved significantly (r ≥ 0.6, p < 0.01) – e.g.: “I feel confident in leading a discussion on the literature presented”, T0: “undecided” (median 3, IQR 2) to T1: “rather agree” (median 4, IQR 1, Z= -5.41, r = 0.85, p < 0.01).

**Discussion:**

The student-led journal club shows to be an effective teaching approach for the field of aging and physical activity within applied health science education. Especially the students’ self-assignment to the studies and involving the scientific community via social media was rated as useful and highly motivating for students and lecturers.

## Background

There is growing knowledge on the effects of physical activity on physical and cognitive functioning in older adults [1–4]. Along with an aging society, academic education in applied health science with a focus on aging and physical activity takes an important role. An exemplary master study program “Sport and Movement Gerontology” (SMG) follows an interdisciplinary academic training strategy that seeks to enable students to gain insights in age-related changes in functioning and develop research skills for analyzing relationships between physical activity and functioning as well as for the development and evaluation of innovative evidence-based exercise-programs for older persons. These exercise-programs may address diverse aspects of functioning and often are implemented in different settings, including geriatric health care.

An evidence-based practice implicates an individual clinical experience and requires a broad knowledge of the current state of research [5]. Healthcare practitioners, clinical researchers and particularly students in applied health science education programs must gain and keep an overview on the increasing number of scientific publications and need to be able to transfer the current evidence into healthcare settings [6, 7]. Journal clubs seek to enable attendees to critically assess the body of evidence, especially the methodological approaches, and the clinical relevance of the findings [8]. Participants receive selected studies prior to a journal club meeting, read and prepare the manuscripts for a discussion. Experts present these studies briefly at a journal club meeting and discuss these in the entire group. Such journal clubs are organized in various health care settings, professional groups, and research groups to facilitate a discussion on current findings [9–11]. Usually, journal clubs are applied as face-to-face meetings or recently also as remote and social media-based approaches [12, 13]. Such technological advances offer the opportunity to involve the authors of the discussed manuscript or external experts in the respective field of research to an interaction in the teaching course. Furthermore, public social media interactions integrated into teaching approaches could enable students to translate their research interests and current topics into plain language and get in touch with interested people or institutions. Such teaching methods also follow the demand to develop, apply and evaluate new means of science communication [14, 15].

Journal clubs are widely used in higher medical education and residents’ clinical teaching, but only little is known on its adaptation, implementation, and evaluation in undergraduate and pre-clinical education [8]. Although most journal clubs are organized and presented by experts on senior levels, student-led journal clubs could be a promising approach to facilitate evidence-based practice [10]. First student-led approaches have been reported, that point to a clear purpose, incentives, and a special training for the role as a journal club leader [9, 10]. Despite the implementation of student-led journal clubs in higher medical education, no investigations on its effects in academic teaching of clinical sport science have been reported yet. Therefore, this project aims to investigate the effects of a student-led journal club on students’ confidence for critical thinking and clinical application skills.

## Methods and materials

### Study design and sample

A teaching evaluation in a pre-post design analysis, with baseline measurement at the beginning (T0) and follow-up measurement after the semester (T1) has been applied within the module “research in clinical health care of older adults” as part of the master study program “Sport and Movement Gerontology” (SMG) at the German Sport University Cologne. Within a clinical teaching cooperation, this course has been conducted in the Department of Geriatric Psychiatry of the LVR-Hospital Cologne (“Landschaftsverband Rheinland”, Regional Council of the Rhineland, Germany). The evaluation period covers the winter semesters of 2018–2021 with 14 scheduled sessions per semester, conducted in the hospital and 2020 during the COVID-19 pandemic via e-teaching.

The seminar is scheduled in the third semester of the master study program. Students have graduated in different bachelor study programs of sport sciences and applied health sciences, e.g., gerontology, physiotherapy, occupational therapy, or nursing science. Relevant differences can be expected from the students’ bachelor background regarding their applied experience in geriatric health care. Students from clinical health sciences, such as physiotherapy or occupational therapy, are expected to have first clinical experience with the target group of older adults through their bachelor programs. In contrast, students graduated in a bachelor of sport sciences probably have only gained little or no clinical experience in geriatric health care. All students assigned to the module have been included in the project after written informed consent for participation in the clinical teaching evaluation.

### Journal club sport and movement gerontology

Embedded into a research-based learning approach of the master study program SMG [16], the primary objective of this clinical module is to enable students to think and act as clinical scientists [7]. Following the methodical and didactical considerations of the “Constructive Alignment” model [17], these key-elements are covered throughout the semester term: clinical insights in ward rounds, staff education programs, geriatric assessment, clinical interventions, and evaluations. Within the exam performance of this module students have to “create an innovative concept for physical activation in healthcare for older adults”.

**Fig. 1:**
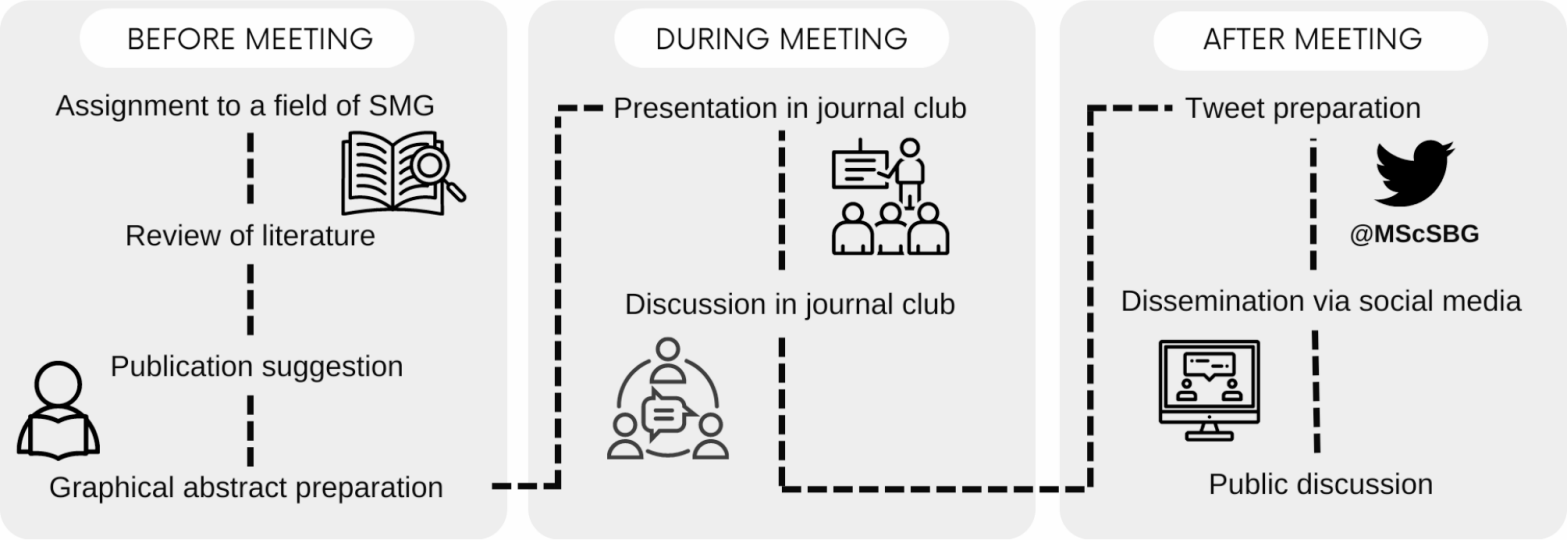
Schedule of the student-led journal club Sport and Movement Gerontology (SMG)

The weekly student-led journal club was implemented to enable students to review, present, discuss and disseminate the current evidence on physical activity in geriatric healthcare. A journal club schedule is illustrated in Fig. 1. In all cohorts, all students had to lead the journal club as key element of the course: Each student assigned him- or herself to a current aspect on physical activity or mobility assessments or exercise interventions in geriatric health care – e.g. physical (in)activity in dementia, endurance training in patients suffering from depression, interrelation between physical activity and sleep in older adults, current innovations in fall prevention, etc. The students conducted a search of the literature on their topic and suggested a current publication which they preferred to present and to discuss in the journal club. All students got access through the learning management system and all attendees were asked to read the publication prior to the next session and prepare aspects for a critical discussion. Within the weekly module teaching sessions, 45 min were scheduled for the journal club. The respective student presented the publication within 10 min via graphical abstract and led a discussion on language understanding, methodological and statistical aspects as well as the transfer of the study results into healthcare and corresponding research gaps.

Together with five key words, recognized as relevant from this study for a concept development, a conclusion of the journal club discussion was communicated together with the student’s graphical abstract via social media (twitter). As part of the university-funded teaching project “#fol2 - linking learning by research and science communication”, the results of the journal club discussion were published via twitter-thread by the SMG-account (@MScSBG). The students’ graphical abstracts were embedded as image and peers as well as authors of the study were tagged in the thread to answer open questions or initiate a further public discussion.

### Instruments & data collection

As part of a standardized teaching evaluation, a questionnaire was used to analyze learning effects of the journal club. Referring to a previous evaluation of a student-led journal club applied in pharmacological education by Donohoe et al. [10], the students rated their confidence to review and summarize the evidence, to present it in a journal club and to lead the journal club discussion. These are the same items and rating scales as used by Donohoe et al. [10]. In addition, the students were asked to rate their confidence in the knowledge of the literature in the field of sport and movement gerontology in addition to their confidence to transfer scientific literature into application-oriented clinical language as well as to develop an evidence-based concept for physical activity promotion in a health care context. All items are presented in Table 2. The baseline measurement at the beginning (T0) and follow-up measurement after the semester (T1) included nine questions regarding their competences using a five-point Likert scale (1–5 points, 1 = excellent, 2 = very good, 3 = quite good, 4 = not so good, 5 = not good at all). Additionally, at T1, a summarizing (total) rating of the journal club with the same Likert scale was applied. Furthermore, the students’ feedback was collected at T1 via semi-structured questionnaire along the following key aspects: “Which topics of SMG should be discussed in future journal clubs?”; “Which types of trials or publications should be addressed?”; “What suggestions do the students have to further improve or adapt the journal club?”. Within an open ended question the students’ free feedback on the journal club was collected at T1 asking: “What is your free feedback on the journal club applied in this semester?“.

### Statistical analysis

For the analysis of the teaching evaluations, median and interquartile ranges, and the Wilcoxon signed-rank test (ordinal scale) were calculated via IBM SPSS Statistics 27.0 (Business Machines, Armonk, NY, USA). Only complete pairs of data were included for pre-post analysis. The effect sizes r were calculated as follows: z-values were divided by the square root of the sample size (N). Values for r < 0.3 are interpreted as small, 0.3 ≤ r ≤ 0.5 as medium, an r > 0.5 as large effects. A significance level of p < 0.05 was set.

## Results

In total 43 students (32 female) were included over the four consecutive semesters. Two students have been excluded from the analysis, as they dropped out of the master study program during the semester, resulting in data of 41 students to be analyzed. Sample characteristics are illustrated in Table 1.

**Table 1:** Sample Characteristics (n = 41, female n = 32 (78%))

	*n (%)*
Age [*years*] Mean (SD) Minimum Maximum	41 25 (1.9) 22 32
Gender	
Female Male	32 (78.0) 9 (22.0)
Education	
Sports Science Gerontology Physiotherapy other health related background	22 (53.7) 5 (12.2) 5 (12.2) 9 (21.8)
Current Semester	
3 5	39 (95.1) 2 (4.9)
Experience in geriatric health care	
Yes No	16 (39.0) 25 (61.0)

The students’ confidence to review and summarize the evidence, to present it in a journal club and to lead the journal club discussion are shown in Table 2. Students’ confidence on participating, leading the journal club and transferring the results into clinical practice improved statistically significant from T0 to T1 (p < 0.01) with large effect sizes ranging between r = 0.60 and r = 0.85. The journal club was rated overall with a median of two points (IQR 1.0), which corresponds to a “very good” rating.

The semi-structured feedback revealed a high agreement with the current topics (n = 10, 31%, 32 in total), and the students suggested to include more clinical trials for patients suffering from depression (n = 5, 16%, 32 in total), clinical exercise trials (n = 5, 16%, 32 in total) and intervention studies in general (n = 4, 13%, 32 in total). Students recommended to discuss all types of studies (n = 9, 32%, 28 in total), agreed with the discussed types of studies (n = 6, 21%, 28 in total) and proposed to assign more randomized controlled trials (n = 5, 18%, 28 in total). The implementation of the journal club was rated as very positive in its current form (n = 6, 33%, 18 in total), five students (28%, 18 in total) reported the discussion should be more efficient and shorter, and three (17%, 18 in total) students endorsed to include more discussion on statistical analyses. The students’ free feedback revealed a high agreement to the journal club’s structure and impact (n = 18, 72%, 25 in total).

**Table 2:** Students’ ratings on their confidence to attend and lead a journal club and to apply the knowledge in health care

Item	T0				T1						
	*n*	Med	IQR		*n*	Med	IQR		Z	*r*	*p*
**Participation in a Journal Club**											
I am aware of the requirements for me as a participant in a journal club.	41	4	1		41	5	0		-4.30	0.67	< 0.01
I feel confident in assessing study designs used in the literature	41	3	1		41	4	1		-4.54	0.71	< 0.01
I feel confident in assessing statistical methods used in the literature.	41	3	2		41	4	1		-3.87	0.60	< 0.01
**Leading a Journal Club**											
I am aware of the requirements for me to lead a journal club	41	4	1		41	5	0		-5.09	0.80	< 0.01
I feel confident in presenting the literature	41	4	0		41	5	1		-4.56	0.71	< 0.01
I feel confident in leading a discussion on the literature presented	41	3	2		41	4	1		-5.41	0.85	< 0.01
**Knowledge and Transfer**											
I have an overview over the evidence in the field of sport and movement gerontology in geriatric health care	41	3	1		41	4	1		-5.16	0.81	< 0.01
I feel confident in transferring findings from articles into application-oriented clinical language	41	3	2		41	4	1		-4.44	0.69	< 0.01
I feel confident in reviewing the current evidence and developing a concept for physical activity promotion in a health care context	41	3	1		41	4	1		-4.86	0.76	< 0.01

Data given as median (interquartile range).

*P*-values given for Mann-Whitney *U* tests.

Effect sizes calculated as *r* (small < 0.3, moderate < 0.5 = moderate, large ≥ 0.5).

T0 = baseline measurement at the beginning of the semester term; T1 = follow-up measurement at the end of the semester term; rating 1 = does not apply; 2 = rather does not apply; 3 = undecided; 4 = rather agree; 5 = strongly agree.

**Fig. 2:**
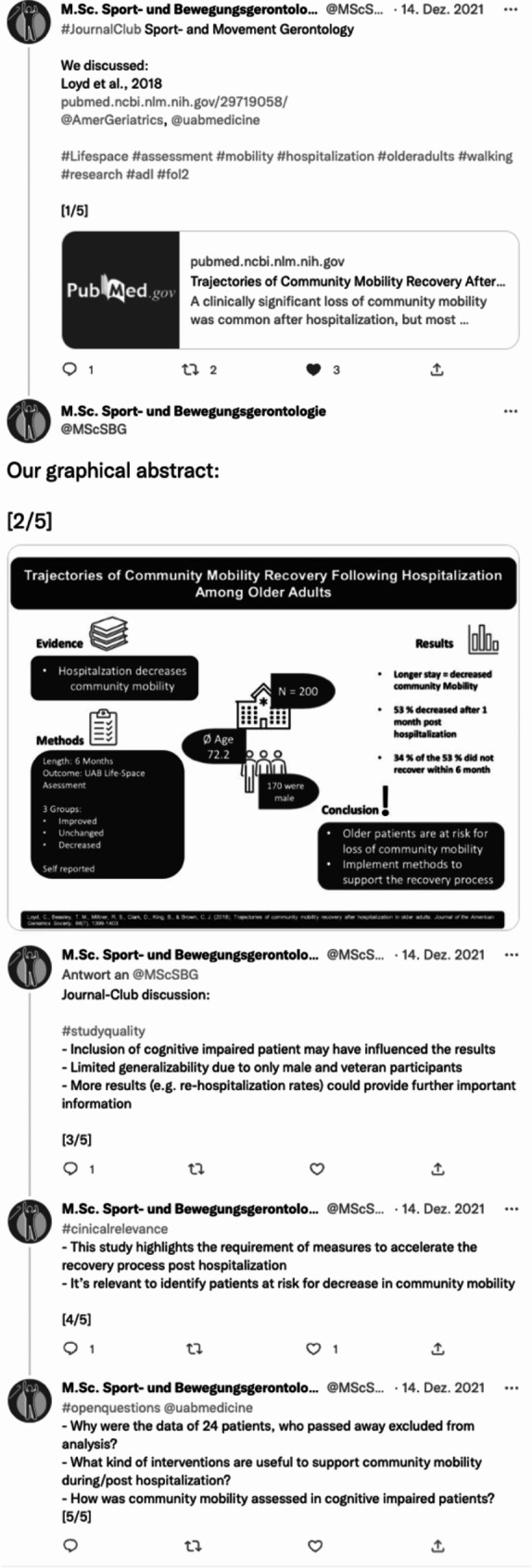
Example of a Twitter thread summing up a student-led journal club discussion

An example for a student’s graphical abstract embedded into a thread is illustrated in Fig. 2.

## Discussion

The aim of this project was to investigate the effects of a student-led journal club on students’ confidence for critical thinking and clinical application skills. With an overall rating of two points representing “very good”, and the significant improvements with large effect sizes r ≥ 0.6 in the students’ ratings on their confidence to attend and lead a journal club (Table 2), the results of this project reveal this student-led journal club as an effective clinical teaching approach. Out of the nine items comparing the students’ confidence at the beginning and the end of the semester, all questions have improved statistically significant by one point in median and showed large effect sizes with r ≥ 0.6. With ratings of “rather agree” (4 points) at follow-up in evaluating study-designs and statistical methods applied in the trials, as well as leading a discussion on the literature presented, the students expressed a relevant improvement, but also indicate further required teaching facilitation. As free feedback, especially the students’ self-assignment to the studies and involving the scientific community via social media were rated as useful and perceived as highly motivating.

The results of this teaching evaluation project are comparable to similar approaches in health science education programs – e.g., reported by Donohoe et al. [10] using the same five-point Likert scale and the analysis revealing statistically significant improvements by one point on the presenters’ confidence to evaluate scientific literature and lead a journal club. Enabling students to prepare, lead and report a journal club, is an important aspect for clinical teaching in applied health science and acts as a milestone for the students’ start in a job position as clinical scientists [7]. As the critical appraisal of scientific literature and especially further applied knowledge transfer skills are key aspects of an evidence-based practice, aspects like the graphical abstract or scientific communication measures could help healthcare practitioners to keep an overview on the current state of research [5].

An interpretation of this project’s results requires to pay attention to relevant study limitations: the pre-post design of the students’ self-assessment could provoke a possible social desirability bias. The study sample predominantly consisted of females, what may have influenced the reported results. Furthermore, the instruments used in this project aimed to assess the students’ self-rated confidence in skills – e.g. the overview of the literature, the presenting performance skills or the knowledge transfer skills, but did not assess the effects of the journal club approach on the skills directly. Within this project, the same 5-point Likert scale was used as reported by Donohoe et al. [10]. To our knowledge, there are no information on quality criteria (e.g. reliability, validity) of the scale available yet. While journal-club approaches are more and more used in clinical teaching, specific assessment tools and an analysis of their psychometric characteristics are still lacking and should be addressed in future research. As this project was embedded into an ongoing module as part of the master study program, no control intervention or control group design was applied. Therefore, it remains unclear whether the reported effects may be due to the journal-club intervention or other educational elements that were applied simultaneously within the course of the module.

Based on our experiences in conceptualizing and implementing a student-led journal club, we see the following developments and perspectives of journal clubs in teaching and health care staff education: Combining activities of scientific communication with a journal club has a potential to disseminate the current evidence and knowledge transfer far further than only to the group of attendees. The presentation of the study by graphical abstract and subsequent communication of the discussion via Twitter are new teaching methods that could increase the visibility and the professional exchange within the field of aging and physical activity. Asking questions and discussion aspects to authors and experts in the field of research using social media, e.g., by using the Hashtag #GeriMedJC, offers a key development that goes beyond traditional journal clubs [12, 18]. Usually, face-to-face journal clubs take place, where the journal club group meets at regular intervals for a discussion. However, results from surveys on the effectiveness of journal clubs revealed the problem of attendance and active participation of the group. Incentives at various levels, such as credit toward work hours, recognition of continuing education credits, or simply the provision of food and beverages, can lead to an increase in attendance and active participation [9]. Referring to the journal clubs’ perspectives suggested by Mark et al. [13], web-based e-journal clubs will enable promising alternatives to traditional face-to-face journal clubs. Technological advances allow to use digital media to present and discuss the state of studies, and simultaneously achieve networking among participants without local presence. Such e-journal clubs are currently implemented as portal-based [19], and social-media approaches [12].

Implementing a student-led journal club and adding temporarily approaches like presentation via graphical abstract and dissemination via social media seems to be very relevant, effective, and future-oriented, leading to high motivation on both sides, students, and lecturers. Especially the improvements in the students’ confidence in participating and leading a journal club during the semester is remarkable. Important to note is that due to the lack of a control-group and the involvement of the module lecturers in the teaching evaluation, results and conclusions should be drawn with caution.

The students’ attained confidence in the knowledge transfer seems to be directly linkable to challenges in their potential role as clinical academics with relevant functions and responsibilities to further develop geriatric health care. Along with the growing knowledge on the effects of physical activity on physical as well as cognitive functioning in older adults [1–4], student-led journal clubs could further contribute to the academic education applied health sciences. Such clinical scientists will play an important role in implementing physical activity as a vital sign in health care and thus will contribute to improve as well as preserve older adults’ independency and quality of life.

## Data Availability

The datasets used and analyzed during the current study are available from the corresponding author on reasonable request.
